# Serum Levels of Selected Cytokines and Chemokines and IgG4 in Children With Recurrent Respiratory Tract Infections

**DOI:** 10.1155/2024/5170588

**Published:** 2024-10-12

**Authors:** Edyta Machura, Helena Krakowczyk, Magdalena Kleszyk, Elzbieta Swiętochowska, Ewa Grzywna-Rozenek, Malgorzata Rusek, Anna Góra, Ewelina Chrobak, Anna Pukas-Bochenek, Maria Szczepanska

**Affiliations:** ^1^Department of Pediatrics, Faculty of Medical Sciences in Zabrze, Medical University of Silesia in Katowice, Katowice, Poland; ^2^Department of Medical and Molecular Biology, Faculty of Medical Sciences in Zabrze, Medical University of Silesia in Katowice, Katowice, Poland

**Keywords:** chemokine, children, cytokines, IgG4, pediatric recurrent respiratory infections

## Abstract

**Background:** Respiratory tract infections are a common health problem. Cytokines/chemokines play a critical role in the regulation of the immune system. Their defective production may predispose to recurrent respiratory tract infections (RRIs), and an excessive immune response may lead to chronic inflammation and cause damage to the respiratory tract. Another biomarker of respiratory infections may be immunoglobulin—IgG4. Its meaning has still been little explored. We wanted to assess the suitability of the levels of biomarkers tested: interleukin (IL)-17A, IL-18, IL-23, normal T cells expressed and secreted (RANTES), and induced protein (IP)-10, as well as immunoglobilun G4 (IgG4) to predict recurrent infections.

**Methods:** The study group (SG) included a total of 130 children (68 girls, 62 boys) between 3 and 17 years of age with RRI. The control group (CG) included 86 healthy children with no symptoms of inflammatory or allergic diseases (44 girls and 42 boys) of the same age. Blood samples were collected in fasting state and then serum samples were frozen and stored until biomarker assay.

**Results:** Serum RANTES, IL-18, IL-23, and IgG4 concentration were higher in all children with recurrent infections vs. those in the CG (*p*  < 0001). Serum levels of IL-17A and IP-10 were also significantly higher in the SG than in the CG, but only in the youngest children. Among the six serum markers, RANTES demonstrated the highest area under the receiver operating characteristic curve (area under curve) value (0.998, 95% confidence interval [CI]: 0.98–1.0, *p* < 0.001) for the diagnosis of RRIs, followed by IL-23 (0.99, 95% CI 0.966–0,999, *p* < 0.001) and IL-18 (0.957, 95% CI 0.921–0.980, *p* < 0.001).

**Conclusions:** RANTES, IL-23, and IL-18 could be strong predictors of respiratory infections recurrence in children.

## 1. Introduction

Respiratory tract infections, particularly in preschool children, are a common health problem. Nursery/kindergarten attendance, short/no breastfeeding period, siblings, low socioeconomic status, poor housing conditions, exposure to tobacco smoke, nonvaccination, male sex, malnutrition, or vitamin deficiency are some of the potential risk factors for recurrent respiratory tract infections (RRIs) [1–3].

Concomitant chronic diseases and immune dysregulation may also determine the rate and severity of respiratory tract infections. Cytokines control inflammatory responses against harmful agents, such as viruses, bacteria, or fungi, synchronizing a variety of processes, including local and systemic immunity control, cell proliferation, and activation of different effector cells, as well as mediation of tissue metabolism and repair [4]. Chemokines are chemotactic cytokines that, through interaction with the corresponding receptors, are involved in the migration patterns of immune cells and influence the production of various cytokines. Their levels may differ according to the severity and location of the infection, and the nature of the pathogen [5]. The excessive immune response may have immunopathological consequences, including damage to the respiratory tract and lungs [6]. Cytokines are considered as biomarkers for many diseases, but, in relation to children with respiratory tract symptoms, they have been little studied and are mainly in acute phase infections. In such a condition, altered cytokine/chemokine profiles were found in children with viral or bacterial infection [6–8]. Immunoglobulin G (IgG) is important in the secondary immune response during infection. Among the four subclasses, IgG4 accounts for approximately 4% of total serum IgG in healthy individuals, and its role in the physiological and pathological immune response has been widely studied. Although low IgG4 concentrations were found in some patients with RRIs, the significance of this finding has not yet been established since low IgG4 concentrations are also confirmed in several healthy children [9].

The study goals included an analysis of serum interleukin (IL)-17A, IL-18, IL-23, regulated upon activation normal T-cell expressed and secreted (RANTES), and induced protein (IP)-10, as well as IgG4 concentrations in children admitted to the General Pediatric Ward for RRIs, and an assessment of the biomarkers tested as predictors of recurrent infections.

## 2. Materials and Methods

### 2.1. Studied Population

The study group (SG) included 130 children (68 girls, 62 boys) aged 3–17 years (median 7.5 ± 4.4 years) referred for recurrent respiratory symptoms to the Department of Pediatrics of the Public Clinical Hospital in Zabrze. The study period lasted from 2013 to 2015. The eligibility criteria for the study included ≥8 respiratory tract infections within a year in children up to 6 years and ≥5 infections within a year in children aged 7–17 years (see details in [4]). All children had been treated with antibiotics during previous infections. The presence of an autoimmune disease or a chronic inflammatory condition was an exclusion criterion for the SG. Most of the children (81%) had risk factors for RRIs. The patients were directly enrolled based on their medical history, physical examination, and baseline laboratory test results, excluding acute infection during the study (complete blood count with differential and C-reactive protein level). Most of the children (81%) demonstrated risk factors for RRIs. The most common clinical diagnosis among the patients was adenoid hypertrophy (AH) (44.62% of the patients, *n* = 58), while the least common was immunodeficiency (abnormal IgG level) (9.23%; *n* = 12), identified in the youngest children (3–6 years). In 48 children (36.92%), asthma and/or allergic rhinitis (AR) were diagnosed. In 40 children with asthma (83.3%), AR symptoms were also present; therefore, children with asthma or AR were treated as a group.

Gastroesophageal reflux disease was diagnosed in 21 children (16.15%), with 12 children having concomitant AH (9.23%) and four with concomitant asthma (3.08%). Forty-five children (34.62%) had some comorbidities. Children with AH were younger (AH: median = 5.0 years [range: 4.0–6.0] vs. those without AH: median = 6.0 years [range: 5.0–14.0]; *p*=0.0025), while children with asthma and/or AR were older (median = 6.0 years [range: 5.0–13.0] vs. median = 5.0 years [range: 4.0–9.0]; *p*=0.031). In 12 patients (9.23%), the cause of RRIs remained undetermined.

The control group (CG) included 86 patients (44 girls and 42 boys), aged 3–17 years (median 8.5 ± 4.7 years) with no history of recurrent respiratory symptoms, with any symptoms of inflammatory or allergic diseases. The children of SG and CG were divided into the following age subgroups: 3–5 years (SG: *n* = 60, CG: *n* = 33), 6–9 years (SG: *n* = 35, CG: *n* = 20), 10–13 years (SG: *n* = 11, CG: *n* = 13), and 14–17 years (SG: *n* = 24, CG: *n* = 20).

Blood samples were collected in fasting state and then serum samples were frozen and stored at −20°C until the cytokine assay. Commercial enzyme-linked immunosorbent assay kits were used for this purpose. IL-17A, IL-23, and IP-10 concentrations were assayed by Diaclone (France) kits. The sensitivity achieved was 2.3 pg/mL; <20 and 5.7 pg/mL, respectively. IL-18 and RANTES levels were measured by Cloud-Clone Corp. (United States) kits (with 5.6 and 0.061 ng/mL sensitivity, respectively). The absorbance readings were made using the μQuant (BioTek, United States), while the results were processed by KCJunior (BioTek, United States). All analytical procedures complied with the manufacturer's recommendations.

The serum IgG4 subclass was assayed with Biding Site reagents (Binding Group Ltd., Birmingham, United Kingdom) and evaluated with a MININEPH Plus machine (Binding Group Ltd., Birmingham, United Kingdom), designed to examine the concentrations of specific proteins in body fluids using the nephelometric measurement technique. MININEPH Plus is a nephelometer that is used to measure light diffusion from antigen/antibody endpoint reactions. The intensity of light scattered by IgG4 immune complexes and specific antibodies in the reagent used is compared to a standard curve drawn prior to test sample measurements. The imprecision (repeatability in a simultaneous series) of the method was 5.1%.

### 2.2. Statistical Analysis

A statistical analysis of selected laboratory values was performed, using licensed Statistica 10.0 software (StatSoft, Poland). Quantitative variables were expressed as a median and a range between the first and third quartiles (25th and 75th percentile). The groups were compared using the Mann–Whitney *U* test. A correlation analysis involved the use of the Spearman's correlation coefficient. Receiver operating characteristic (ROC) curve analysis was performed to evaluate candidate indicators with regard to the evaluation of children with RRIs. The Youden index method was used to determine cutoff points. *p* values < 0.05 were considered statistically significant.

According to the Declaration of Helsinki, the subjects and their parents/legal guardians were informed of the research purpose, its nature, and the applied methods. Written informed consent forms were obtained from the parents or legal guardians of the children or from the subjects themselves when aged ≥16 years.

The study was approved by the Ethics Committee of the Medical University of Silesia, Katowice, Poland (No. KNW/0022/KB1/77/14).

## 3. Results

Table 1 for laboratory results in children with SG CG. The serum concentrations of RANTES, IL-18, IL-23, and IgG4 were higher in all children with recurrent infections vs. those of the CG (*p*  < 0.001) (Table 2).

Serum levels of IL-17A and IP-10 were also significantly higher in SG children than in CG subjects, but, after stratification by age matched, increases in IL-17A and IP-10 levels were found only in the youngest group (*p*=0.005, *p*=0.01, respectively).

Serum levels of IL-18 were positively correlated with IgG4, IL-17A, and IL-23, (*r*: 0.53, *p*  < 0.001, *r*: 0.34, *p*  < 0.001, and *r*: 0.7, *p*  < 0.001, respectively). The latest (IL-23) was positively correlated with IgG4 and IL-17A (*r*: 0.38, *p*  < 0.001, *r*: 0.5) and negatively with IP-10 (*r*: −23, *p*  < 0.006). RANTES were positively correlated with IL-23, IP-10, and IL-17A (*r*: 0.24, *p*=0.004, *r*: 0.35 *p*  < 0.001, and *r*: 0.74 *p*  < 0.001, respectively) and negatively with IgG4 (*r*: −0.3, *p*  > 0.0021). IP-10 was positively correlated with IL-17A (*r*: 0.35, *p*  < 0.0001) and negatively with IgG4 and IL-23 (*r*: −0.31, *p*  < 0.0002, *r*: −0.23, *p*=0.006) and IL-17A negatively with IgG4 (*r*: −0.21, *p*  < 0.01).

Serum levels of IL-17A (AH+: 28.5 [24.7; 31.6] vs. AH−: 31.1 [27.4; 35.6] pg/mL) and RANTES (AH+: 732 [7211; 7631] vs. AH−: 7560 [7330; 7681.5] pg/mL) were significantly lower in children with AH than in those without AH (*p*=0.03, *p*=0.007, respectively).

No differences were found in the concentrations of the parameters assessed between children with asthma/AR, immunodeficiency, and those without the symptoms mentioned above.

### 3.1. The Predictive Value of Selected Immunological Parameters for the Risk of RRI in Univariate Analysis and Logistic Regression Analysis

To assess the relationship between the concentration of selected immunological parameters and the risk of respiratory tract infection in children, a univariate analysis was used, including the evaluation of the odds ratio and the 95% confidence interval. The risk of RRI was shown to be associated with higher concentrations of IgG4 (OR: 0.996; 0.995–0.998, *p*  < 0.001), IL-17A (OR: 0.94; 0.89–0.98, *p*  < 0.0009), IL-18 (OR: 0.87; 0.84–0.90, *p*  < 0.000001), IL-23 (OR: 0.93; 0.90–0.96, *p*  < 0.0001), and RANTES (OR: 1.02; 1.00−1.0; *p*  < 0.0001).

A logistic regression analysis showed the following independent predictors of RRI: IL-18 (log OR: 1.11; 1.05–1.18; *p*  < 0.0002) and IgG4 (log OR: 1.05; 1.02–1.07, *p*  < 0.004).

### 3.2. ROC Curves to Predict RRI

**Figure 1:**
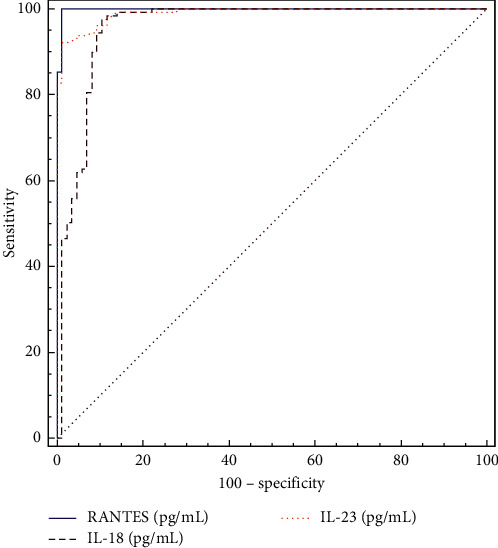
Comparison of ROC curves of RANTES, IL-18, and IL-23, for predicting occurrence of respiratory tract infection. IL, interleukin; ROC, receiver operating characteristic.

Among the six serum markers, RANTES demonstrated the highest area under curve (AUC) value (0.998, 95% CI: 0.98–1.0, *p* < 0.001) for the diagnosis of RRI, followed by IL-23 (0.99, 95% CI 0.966–0.999, *p* < 0.001) and IL-18 (0.957, 95% CI 0.921–0,980, *p* < 0.001) (Table 3) (Figure 1).

## 4. Discussion

Our study showed a significant systemic elevation of all examined pro-inflammatory cytokines/chmokines IL-17A, IL-18, IL-23, RANTES, and IP-10 during disease-free periods in the cohort of children with RRIs, which may indicate their constant release. The analysis of the ROC curve showed that RANTES, as a biomarker associated with RRI, demonstrated the highest sensitivity and specificity (100% and 98.5%, respectively; the cutoff point of >6843) among the parameters studied. RANTES (the C–C chemokine ligand5-CCL5) belongs to CC chemokines, which are potent chemoattractants for natural killer, memory T, and dendritic cells, as well as for monocytes and eosinophils. It is involved in the activation of T cells and the release of pro-inflammatory cytokines [10, 11]. The production of RANTES is induced by various viruses, including adenovirus, coxsackie virus, rhinovirus (RV), respiratory syncytial virus (RSV), coronavirus, and influenza virus, as well as by bacteria, including Streptococcus pneumonia—a major respiratory pathogen [11, 12]. Some data suggest that CCL5 is an essential factor for the induction and maintenance of protective pneumococcal immunity [13]. Furthermore, decreased expression of CCL5 and elevated viral load of severe acute respiratory syndrome-coronavirus-2 (SARS-CoV-2) in nasopharyngeal samples were associated with poor outcomes of coronavirus disease 2019 (COVID-19) [14]. RANTES promotes the T helper1 (Th1) and T helper2 (Th2) response, and its increased secretion could contribute to airway inflammation and obstruction. Furthermore, it is suggested that high levels of RANTES during acute RSV bronchiolitis may be predictive of the later development of recurrent wheezing [15]. In our study, RANTES was correlated with other cytokines, except IL-18.

IP-10 was elevated exclusively only in the group of the youngest children. ROC curve analysis showed that IP-10, as a parameter associated with RRI, showed the lowest sensitivity and specificity among the biomarkers evaluated.

IP-10 is induced in a variety of cells in response to Th1 cytokine interferon gamma (IFN-*γ*) and also by bacterial products and viruses. In addition to its chemotactic function, IP-10, like other chemokines, exhibits direct antimicrobial activity against some bacteria, including *Streptococcus pneumoniae* [16]. Recent studies suggest that serum IP-10 level may be an important biomarker for various viral infections, including upper airway infection with RV, RSV bronchiolitis, bacterial pneumonia, tuberculosis in children, and SARS-CoV-2 infection [8, 17].

Elevated levels of IL-18 were confirmed in all groups of patients. In addition, this cytokine, as the ROC analysis showed, revealed high sensitivity and specificity to predict RRI. IL-18 is a pro-inflammatory cytokine, belonging to the IL-1 family, which controls Th1 and Th2 responses. Furthermore, IL-18 stimulates Th17 differentiation and IL-17 production. IL-18 is responsible for the induction of adaptive immune responses after innate immune responses, and it is assumed to play an important role in anti-infection defence [18]. IL-18 protects against RV-induced colds and asthma exacerbations [19] and plays an important role in the early antibacterial host response during pneumococcal pneumonia and *Streptococcus agalactiae* infection [20]. Elevated levels of IL-18 were identified in bronchiolitis [6], recurrent tonsillitis [21], refractory Mycoplasma pneumianiae [22], and in patients with SARS-CoV-2 infection. IL-18 might protect against COVID-19, but IL-18 hypercytokinemia is associated with an increased severity of COVID-19 [23].

In our study, serum IL-23 concentrations were elevated in all age groups, while IL-17A concentration was elevated only in the youngest patients, although their concentrations were positively correlated with each other. Although the concentration of IL-23 in the ROC analysis was characterized by high sensitivity and specificity, IL-17A showed the lowest specificity, compared to the other parameters analyzed. Along with other cytokines, the IL-23/17 axis plays a central role in the development of inflammation and in host defense against bacterial infections. IL-17-dependent neutrophil-mediated protection was also observed during *Streptococcus pneumonia*, *Klebsiella pneumoniae*, and *Pseudomonas aeruginosa* infection [24, 25]. Recent studies have described an emerging role for IL-17 in protecting against intracellular pathogens, such as *Listeria monocytogenes* or *Mycoplasma pneumoniae* [26, 27]. The secretion of defective IL-17 was confirmed in fungal infections [28]. On the other hand, IL-17 plays a detrimental role in the pathogenesis of *P. aeruginosa* airway infection during acute exacerbations of chronic obstructive lung disease [29]. The character of IL-17 during viral infections remains controversial, as IL-17A can be protective or pathogenic, depending on specific circumstances [30, 31]. High levels of IL-17 were associated with COVID-19 severity [31] and long COVID-19 [32].

All examined cytokines are pleiotropic and are involved in both the innate and adaptive immune responses. They also play an important role in various inflammatory, autoimmune, and allergy diseases, while autoimmune diseases were excluded in our SG children. No differences were observed in the concentrations of the parameters assessed between the children with and without atopic diseases.

Low serum IgG4 is rare but can be associated with recurrent respiratory, urinary tract infection, allergies, chronic diarrhea, and fungal infections [33, 34]. In our study, IgG4 concentrations were significantly higher in the SG than in healthy children. ROC analysis showed IgG4 with a low sensitivity but as a highly specific marker for RRI. An IgG4 synthesis (like IgE) depends on Th2 activation; however, IgG4 does not participate in allergic sensitization. IgG4 shows anti-inflammatory activity and plays a role in induction of allergy tolerance [35]. Elevated serum IgG4 levels are significantly higher in helminthiasis infections, in adults with recurrent infection, in cystic fibrosis, severe asthma, atopic, and autoimmune diseases, as well as in IgG4-related diseases. Specific serum IgG4 is used as a marker of tolerance in allergic diseases and during immunotherapy. Hypergammaglobulinemia of IgG4 occurs in 5% of the healthy population and appears to have no consequences [34]. In our study, IgG4 concentration was correlated, among others, with IL-18, which stimulates Th1 and Th2 cells and therefore may have at least partially influenced IgG4 synthesis.

A limitation of the current study is that only pro-inflammatory cytokines were measured, while, as we know, the balance between pro- and anti-inflammatory cytokines plays an essential role in the control of the inflammatory response and, consequently, in the resolution of respiratory infections. The advantages of this study include unselected children, diagnosed in the order of admission from the primary care.

## 5. Conclusions

Our results suggest that cytokines are constantly triggered in children with RRI. RANTES, IL-23, and IL-18 could be strong predictors of RRI. Children with RRI showed higher levels of IgG4, which may have reflected continuous exposure to antigens (viruses, bacteria, and allergens) and may have played a role as a humoral inhibitory factor.

## Figures and Tables

**Table 1:** Laboratory findings in the SG and CG children.

Parameter	SG (*n* = 130)	CG (*n* = 86)	*p*
Sex (girls/boys)	*n* = 68/62, 52%/48%	*n* = 44/42, 51%/49%	—
Age (years)	6.0 (4.0; 10.0)	6.0 (5.0; 13.0)	0.16
Leukocytes (10^3^/*µ*L)	7.5 (5.9; 9.1)	6.4 (5.3; 7.7)	0.005
CRP (mg/L)	0.67 (0.3; 1.3)	0.86 (0.44; 1.54)	0.197
Eosinophils (%)	2.0 (1; 4.0)	3.0 (2.0; 3.0)	0.9
Eosinophils (*µ*L)	198 (102; 286)	166 (104; 208)	0.3
IgA (g/L)	1.1 (0.7; 1.55)	0.9 (0.67; 1.15)	0.1
IgM (g/L)	0.96 (0.72; 1.3)	0.9 (0.67; 1.15)	0.28
IgG (g/L)	8.94 (7.8; 10.8)	9.6 (8.4; 10.9)	0.13
IgE (IU/mL)	44.6 (13.7; 123.2)	31.78 (18.0; 72.1)	0.34

*Note*: Data expressed as the median (*Me*) and the 25th and 75th percentile (Q1; Q3).

Abbreviations: CG—control group; CRP—C-reactive protein; IgA, IgE, IgG, IgM—immunoglobulin classes: A, E, G, and M; SG—study group.

*p* test Mann–Whitney.

**Table 2:** Serum cytokines/chemokines and IgG4 in children with RRI (SG) and CG.

Children with recurrent infection (SG)		CG	
IL-17A (pg/mL)			

All (years)	30.1 (25.7; 35.1)	All (years)	26.75 (20.82; 31.9)^$^
3–5	28.70 (25.35; 35.00)	3–5	23.90 (20.74; 30.10)^$^
6–9	28.60 (25.10; 31.20)	6–9	25.80 (22.60; 31.66)
10–13	35.20 (28.80; 36.20)	10–13	27.43 (19.83; 36.51)
14–18	32.35 (27.00; 35.75)	14–18	29.07 (21.97; 36.45)

IL-18 (pg/mL)			

All (years)	173.12 (162.31; 191.76)	All (years)	140.00 (128.60; 148.70) ^*∗*^
3−5	178.11 (163.21; 192.38)	3–5	145.20 (138.10; 151.30) ^*∗*^
4–9	163.70 (161.32; 177.50)	6–9	141.40 (136.95; 149.20) ^*∗*^
10–13	191.27 (188.72; 193.68)	10−3	131.70 (119.20; 139.80) ^*∗*^
14–18	188.74 (161.88; 191.76)	14–18	129.80 (126.80; 137.20) ^*∗*^

IL-23 (pg/mL)			

All (years)	808.5 (746.0; 927.0)	All (years)	596.5 (498.0; 663) ^*∗*^
3–5	810.5 (758.5; 933.5)	3−5	563 (467; 612) ^*∗*^
6–9	772 (741; 818)	6–9	507 (484; 622) ^*∗*^
10–13	956 (829; 967)	10–13	598 (538; 693) ^*∗*^
14–18	821.5 (731.5; 936)	14–18	663 (602.5; 694.5) ^*∗*^

IP-10 (pg/mL)			

All (years)	73.1 (69.2; 75.4)	All (years)	66.75 (61.5; 77.4)^#^
3–5	72.75 (69.25; 74.75)	3–5	65.1 (59.8; 73.8)^%^
6–9	74.8 (68.7; 79.5)	6–9	64.9 (60.9; 79.9)
10–13	70.3 (69.2; 72.3)	10–13	73.3 (66.2; 79.7)
14–18	74.3 (69.65; 75.7)	14–18	69.4 (64.4; 80.35)

RANTES (pg/mL)			

All (years)	7493 (7245; 7658)	All (years)	5376.5 (4582; 6120) ^*∗*^
3–5	7427.5 (7221; 7643)	3–5	4924 (4070; 6062) ^*∗*^
6–9	7452 (7254; 7631)	6–9	5358.5 (4867; 6175.5) ^*∗*^
10–13	7681 (7432; 7763)	10–13	5891 (5321; 6223) ^*∗*^
14–18	7561 (7339; 7670)	14–18	5597.5 (4876; 6226.5) ^*∗*^

IgG4 (mg/L)			

All (years)	499.15 (400.30; 589.20)	All (years)	290.05 (189.70; 395.10) ^*∗*^
3–5	483.30 (399.55; 581.75)	3–5	371.80 (284.10; 417.30) ^*∗*^
6–9	466.80 (403.20; 604.30)	6–9	353.40 (238.90; 383.80) ^*∗*^
10–13	527.60 (401.10; 587.90)	10–13	206.80 (178.10; 277.80) ^*∗*^
14–18	512.20 (395.70; 593.80)	14–18	169.65 (136.45; 310.35) ^*∗*^

Abbreviations: CG, control group; SG, study group.

^*∗*^*p* < 0.0001, ^$^*p* < 0.005, ^#^*p* < 0.009, and ^%^*p* < 0.01.

**Table 3:** ROC curves analysis of selected parameters as predictors of the occurrence of recurrent infection.

Parameter	Cutoff value	Sensitivity (95% CI)	Specificity (95% CI)	AUC (95% CI)	*p* value
RANTES (pg/mL)	>6843	100 (97.2–100)	98.84 (93.7–100)	0.998 (0.98–1.00)	<0.0001

IL-18 (pg/mL)	>155.1	97.6 (93.4–99.5)	89.5 (81.1–95.1)	0.957 (0.921–0.980	<0.0001

IL-23 (pg/mL)	>724	91.5 (85.4–95.7)	98.8 (93.7–100.0)	0.990 (0.966–0.999)	<0.0001

IgG4 (mg/L)	>452	60.0 (51.0–68.5)	97.67 (91.9–99.7	0.89 (0.84–0.928)	<0.0001

IL-17A (pg/mL)	>29.9	84.6 (77.2–90.3	45.3 (34.6–56.5)	0.618 (0.549–0.683)	0.0053

IP-10 (pg/mL)	>67.8	83.1 (75.5–89.1)	55.8 (44.7–66.5)	0.605 (0.537–0.67)	0.0159

Abbreviations: 95% CI—lower and upper limits of the 95% confidence interval; AUC—area under curve; IP-10—IFN-*γ*-inducible protein-10; RANTES—regulated upon activation normal T-cell expressed and secreted; ROC—receiver operating characteristic.

## Data Availability

The data used to support the findings of this study are available from the corresponding author upon request.
